# Degradation of Bioderived Polyurethane Composites by Spectroscopy in ISO20200 Composting Conditions

**DOI:** 10.3390/polym16142071

**Published:** 2024-07-20

**Authors:** Alexander Caschera, Tristan Calayan, Nicola Piccolo, Adel Kakroodi, Jason James Robinson, Guerino Sacripante

**Affiliations:** 1 Department of Chemistry & Biology, Toronto Metropolitan University, 350 Victoria Street, Toronto, ON M5B 2K3, Canada; caschera@mcmaster.ca (A.C.); tristan.calayan@gmail.com (T.C.); nicola.piccolo@torontomu.ca (N.P.); 2 Evoco Ltd., 661 University Ave., Toronto, ON M5G 1L7, Canada

**Keywords:** bioderived, sustainable polymers, biodegradation, polyurethane

## Abstract

Polyurethane foam compositions derived from bioderived polyester polyols with various additives were evaluated for disintegration under composting conditions using the ISO 20200 standard and were characterized by thermogravimetric analysis, microscopy, infrared spectroscopy, and imaging to provide additional insight. Compared to polyether polyol-based polyurethanes, the bioderived polyurethanes were found to display increased disintegration with an average mass loss of 25.4 ± 3.6 weight percent when subjected to composting conditions for 45 days, suggesting that these materials are less likely to persist in the environment when compared to other types of commodity plastics. Additives such as carbon black and lignin added within the foam composition did not accelerate the disintegration.

## 1. Introduction

Plastics have become an integral part of the operation of modern society in the past century, and they are predominantly manufactured from non-renewable fossil fuels and inevitably discarded into the environment. Most plastics are resistant to biodegradation and have resulted in the accumulation of micro- and nano-plastic particles with adverse health effects on both sea and land animals. Their resistance to biodegradation is mainly attributed to their macromolecular structure and hydrophobicity, which limit the ability of the microorganisms to transport themselves into cells for metabolic disintegration; the biodegradation relies on extracellular enzymes to achieve some limited depolymerization [1]. This has led to the development of biodegradable plastics such as polyhydroxyalkanoates (PHAs), which structurally comprise ester linkages in the polymer backbone that are easily hydrolyzed by microorganisms to monomeric metabolites. PHAs have evolved in nature via numerous microorganisms which utilize them as storage energy sources through depolymerization [2].

However, the emergence of commercial PHAs, such as polyhydroxy butyrate, copolymers, and other biodegradable polyester-based plastic alternatives, have had little success due to their inferior mechanical properties and higher cost compared to traditional polymers. In 2021, the production of these polyesters represented less than 0.1% of all commercial polymers [3] and was mostly driven by government procurement policies and public awareness of sustainability [4].

Polyurethane-based plastics are an important class, representing about 6.6% of all polymers produced globally [3], with broad applications in construction, the automotive industry, furniture, footwear, insulation, coatings, adhesives, elastomer foams, and consumer goods. These include thermoplastic as well as rigid and flexible foams, of which the latter accounts for 67% of all polyurethane produced [5]. Polyurethanes are produced from the polymerization reaction between polyols and diisocyanates in the presence of chain extenders, blowing agents, surfactants, fillers, plasticizers, and colorants. Blowing agents create a polyurethane foam, while surfactants control the bubble formation and, therefore, the cell formation of the foam. In general, fillers increase stiffness, plasticizers enhance malleability, and pigments add color to the material. The polyols utilized for polyurethanes are typically of low molecular weight and can be either a polyether derived from the polymerization of alkylene oxides or hydroxyl-terminated polyester resin.

As polymeric materials are subjected to differing environmental factors, including temperature changes; pH changes; exposure to water, light, and oxygen; and physical stress, they tend to undergo physical and chemical changes that discolor and fracture the bulk material. Generally, these changes affect all materials over time; however, the rate at which any one of these factors can degrade a specific polymer varies based on the material’s susceptibility to these erosive forces [6,7]. As an example, polyesters are formed from multiple ester bonds, which are known to be susceptible to enzyme-catalyzed hydrolysis [8,9]. Ultimately, polymeric materials are converted into increasingly smaller and more reactive species over time, but this process can lead to the intermediate production of microplastics which can cause harm to living species and the environment [10]. Also, since these changes impact functional structures that comprise the backbone structures of these polymers, the degradation of these components can be visualized by infrared spectroscopic methods [11].

Polymers and other materials can be subjected to biodegradation by decomposing microorganisms within the same environment. Enzymes, acids, and other reactive chemical species are exuded to assist in harvesting nutrients and organic molecules so that they can be used by the present microbiota [12]. For carbon-containing polymers and materials, this ultimately results in the production of volatile carbon compounds, including carbon dioxide and methane, which results in a decrease in organic carbon within the environment over time [13].

Our interest is in the development of a more sustainable polyurethane composite which is biodegradable and derived primarily from bioderived components, especially polyester polyols, plasticizers, and fillers, as well as colorants and diisocyanates. Determining the biodegradation of plastic materials under controlled composting conditions, can be achieved by using the ASTM D5338, where the plastic product must demonstrate a satisfactory rate of biodegradation by achieving an acceptable ratio of conversion to carbon dioxide within 180 days. However, this test is quite time-consuming and expensive to carry out on a routine basis as the set-up can only be used on an individual sample. There is another ASTM test, the ISO 20200 standard, which can more easily measure the disintegration of plastics in 45 days and allow more samples to be carried within an equipment set-up. This latter test is extremely useful when developing polyurethane formulations with various components as a screening method for an indication of biodegradation by further evaluating and characterizing disintegration components through imaging, gravimetric analysis, infrared spectroscopy, and optical microscopy. The current work examines various formulations of bioderived polyurethane–polyester foam composites to assess the drivers of easier disintegration in the composting environment and provides an accelerated methodology for identifying markers of biodegradation prior to committing to a resource-intensive full biodegradation study.

## 2. Materials and Methods

### 2.1. Biobased Polyurethane Foam Formulation

The material stocks used for samples 2 to 8 were prepared according to Example 9 of US Patent 10,934,384, titled “Polyurethane elastomer compositions, and processes thereof”.

**Scheme 1:**
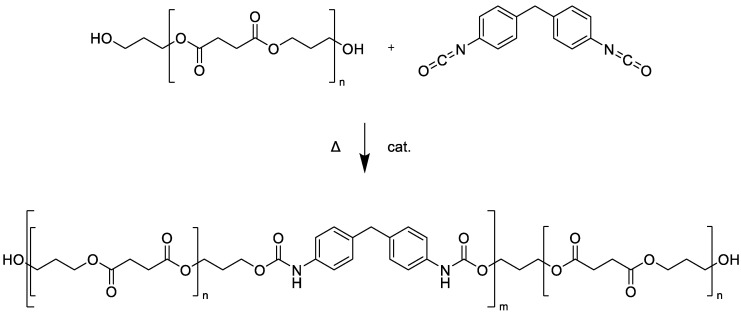
General reaction equation for polyester-based polyurethane polymeric backbone supplied by Evoco Ltd.

General procedure: 35 g of (1,3-propylene-succinate) was combined with 10.5 g of tributyl citrate, 0.19 g of TEGOSTAB^®^ (Evonik, Essen, Germany), 1.03 g of molten 1,3-propanediol, 0.37 g of 1,4-diazabicyclo[2.2.2]octane solution (DABCO 33-LV; Evonik), 0.32 g of water, 0.035 g of diethanolamine, and 1.24 g of FATE® dye (Bao Shen Polyurethane Tech, Hong Kong, China) within a 200 mL plastic container under overhead stirring and was heated to 50 °C. To this container, 11.9 mL of methylene-diphenyl diisocyanate (Suprasec 2379; Huntsman Corp., Arlington, TX, USA)was quickly added to the mixture and allowed to stir for 5 s before removing the container from the mixer and allowing the reaction to proceed (Scheme 1). After 6 min of resting, the hardened foam was demolded from the container and evaluated to determine whether the foam conformed to a density of 0.15 g/cm^3^ and had an Asker C hardness of 17. [14] The weight percent additions of kraft lignin (West Frazer Mill Ltd, Vancouver, Canada), coconut charcoal (Sigma-Adrich, St.Louis, MO, USA), biobased 1.5-pentamethylnendiisocyanate (Mitsui Chemical Corp., Tokyo, Japan), glycerol (Bio Basic Inc, Markham, Canada), and *Bacillus* spp. probiotics (Evoco Ltd., Toronto, Canada) are listed in Table 1. Additional information regarding the included bacterial adducts can be found under PCT WO2021102589 [15] and the biobased additives can be found under PCT WO2022126242 [16].

### 2.2. Sample Preparation

These samples were made in accordance with the ISO 20200 specifications [17]. The polyurethane material stocks required to make each set of samples were supplied by Evoco Ltd. Many of these polyurethane foam compositions were derived from biobased poly (1,3-propylene-succinate), biobased tributyl citrate plasticizer, and methylene diphenyl diisocyanate, as described in Example 9 of US Patent 10,934,384; the fillers, colorant, and bacteria are listed in Table 1 [14]. A commercially sourced thermoplastic polyurethane, WHT-8885 (Wanhua Chemical Group Co. Ltd.; Yantai, China), which consists of an MDI-polyether backbone, was used for comparison [18]. These materials were cut with a stainless steel razor blade into 15 mm × 15 mm × 5 mm. The samples were weighed into 10 g batches, placed into a Model 48 Fisher vacuum oven (Fisher Scientific Co.; Hampton, NH, USA), and allowed to dry over a 24-h period before reweighing. Thirty seconds before placing the samples into their respective composting reactors, the reweighted sample batches were dipped and soaked in a container filled with distilled water. Extended trial samples were cut into larger 15 mm × 15 mm × 15 mm cubes and split into 2 5 g batches and are included in the Supplementary Materials.

### 2.3. Synthetic Waste Preparation

The synthetic waste used to degrade the foam samples under thermophilic composting conditions was made immediately before use with the sets of prepared samples. In a large pail, 1 kg of synthetic waste was combined, prepared, and mixed for each reactor used during the trial. An additional 0.5 kg of excess synthetic waste was also made per trial. This was completed by adding the corresponding dry components in the following percentages: 40% sawdust/animal bedding (Pet Valu Canada Inc.; Markham, ON, Canada); 30% alfalfa rabbit feed pellets (Martin Mills Inc., Elmira, ON, Canada); 10% municipal compost (All Treat Farms Ltd.; Arthur, ON, Canada); 10% cornstarch (ACH Food Companies, Inc.; Oakbrook Terrace, IL, USA); 5% sugar (Redpath Sugar Ltd.; Toronto, ON, Canada); 4% corn oil (Mazola Corn Oil; ACH Food Companies, Inc.; Oakbrook Terrace, IL, USA); and 1% urea (Alpha Chemicals; Cape Giradeau, MO, USA). After the addition of the dry components, distilled water was added until it constituted 55% of the total synthetic solid waste mass, and the components were mixed by mechanical agitation of the pail for 15 min. Prior to use, the pH and nitrogen content were measured using a pH meter (cat# STARA2117, Thermo Fisher Scientific; Waltham, MA, USA) and a coulometric soil testing kit (Lustre Leaf Products, Inc.; Atlanta, GA, USA). If the nitrogen was determined to be deficient, the urea addition was doubled and mixed into the synthetic waste 1 h before retesting the nitrogen content. Synthetic waste samples were taken from the initial excess waste and from the individual reactors following the trials by individually labelling and filling 50 mL conical polypropylene tubes and storing them at −4 °C until analysis could be performed after the trial.

### 2.4. Composting Reactor Setup

Lidded plastic polypropylene containers measuring 346 mm (l) × 210 mm (w) × 124 mm (h) were used (cat# 16428012, Sterilite; Townsend, MA, USA) as sample reactor vessels. To these containers, 2 5 mm holes were drilled 65 mm from the container bottom, vertically centered on the 200 mm (w) sides. These containers were filled with 1000.0 g of premixed synthetic waste. The prepared samples were then mixed in weighted batches into the synthetic waste reactors. The initial reactor weights were recorded and then loaded into a 58 °C incubator (cat# 51030515, Thermo Fisher Scientific; Waltham, MA, USA) equipped with a 1 L/min external aquarium air pump (cat# 77846, Spectrum Brands Pet, LLC.; Blacksburg, VA, USA) to provide fresh air. Within 30 days, the sample reactors were mixed and rehydrated to 100% initial weight following a standard schedule, as specified in ISO 20200. Thirty days after the incubation started, the reactors were mixed and rehydrated to 80% initial weight according to the schedule. Throughout the trial, the reactor vessels were repositioned within the incubator to prevent localized variations within the incubator from affecting reactor decomposition.

### 2.5. Sample Collection and Evaluation

After 45 days of reactor upkeep and incubation, the sample batches were individually sifted and removed from the composted synthetic waste. The collected materials were exhaustively rinsed with distilled water until there was an absence of floating particulate matter. The sample batches were then placed in a vacuum oven at 60 °C to dry until the sample mass change was less than 1% of the initial weight, with a drying time of around 3–5 days. Clean, dried sample batches were then weighed to determine the degree of disintegration of each set compared to the composting period. Additionally, the samples were imaged using a Nikon Eclipse E200 upright microscope (Nikon Co., Ltd.; Tokyo, Japan) at 40× magnification and characterized by Fourier transform infrared spectroscopy using an Agilent Cary 630 equipped with a diamond attenuated total reflectance accessory (Agilent Technologies, Inc.; Santa Clara, CA, USA). For an additional extended-period trial, a 10 g batch of samples was split into 3 identical 5 g sets, where 1 experienced the standard 45-day trial and the other 2 were maintained in composting conditions until 75 and 90 days of composting had elapsed, respectively.

### 2.6. Soil Analysis

Soil samples were taken from the synthetic solid waste when it was first prepared and from each reactor immediately following sample collection. These soil samples underwent thermogravimetric analysis (TGA) using the TA Instruments TGA55 (Waters Corporation; Milford, MA, USA), which is equipped to determine the decrease in volatile organic solid components, indicating composting activity. Approximately 10 mL of frozen synthetic waste sample was placed into a pestle and mortar before being cryogenically frozen with liquid nitrogen and crushed into a fine powder. An excess of 50 mg of powdered synthetic waste was then loaded onto a 100 μL platinum pan and placed into the TGA sampler. The thermal profile used to test the volatile organic solids content included a 20 °C/min ramp to 105 °C, which was maintained for 1 h before ramping by 20 °C/min up to 550 °C and holding for 6 h. Both isotherm periods were set to abort and to proceed if the change in mass of the sample was less than 0.5%/min to reduce excessive heating.

## 3. Results and Discussions

### 3.1. Sample Mass Loss during Composting

Under the scope of this work, a 45-day trial was run for each of the sample sets. Of these sample sets, there were four controlled additive variations of the polyester-based polyurethane created and supplied by Evoco Ltd. (Toronto, ON, Canada) and one polyether-based thermo-polyurethane control set 1 supplied by Wanhua Chemical Group Co., Ltd. (Yantai, China). The samples provided by Evoco Ltd. are based on existing and developmental product lines. This formulation of the polyurethane material was produced using a general package of polyol and diisocyanate, and additives were used to create a polyurethane material that could be processed into sample pieces. From the control polyester-based polyurethane sample 2, modifications to the additive package were made to determine whether they influenced the rate of degradation under composting conditions. These modifications included using biobased fillers and plasticizers and replacing petroleum-based diisocyanate with a biobased alternative. The full list of modifications can be seen in Table 1.

The ISO 20200 trial itself is designed to simulate the conditions found within an industrial composting operation to determine whether a material will suitably degrade into compost for agricultural use [19]. Samples are loaded into a synthetic waste mix before being aerobically agitated, periodically rehydrated, and incubated at thermophilic conditions using a schedule set out by the standard. This test is advantageous since the materials and equipment required to run the trial are not necessarily specialized, and the test can be easily performed in an industrial setting, although some additional analyses can be performed after the trial to determine key factors that may contribute to the degree of degradation observed. 

**Figure 1:**
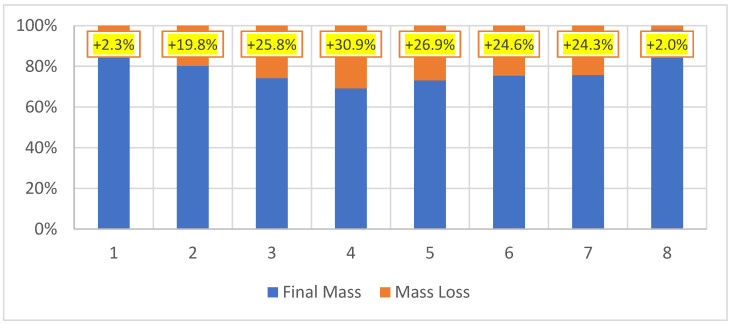
Aggregate sample mass loss from the standard ISO 20200 degradation trial. Samples were handled in accordance with ISO 20200 for 45 days. The lower, blue portion represents the final mass of the sample as a percentile of mass remaining after the trial. The upper, orange portion and the data callout on the graph represent the percentage mass lost during the trial. Sample set numbers are assigned according to Table 1.

On average, the mass loss of the tested samples was 25.4 ± 3.6%, excluding sample sets **1** and **8**, which lost 2.0% and 2.3% of their initial mass. Regarding the degradation performance of the formulation sets and the commonly shared additive modifications, the dye-less sets experienced an exceptional average mass loss of 28.6 ± 2.5% (Figure 1). This suggests that the addition of biobased lignin and charcoal fillers to the polyurethane material increased the degradability of the material. 

A comparison was made with the results obtained by Kupka et al. [20] when evaluating poly hydroxybutyrate–polyurethane composites; they observed mass losses of 2.1% for their control, 0.7% for their commercial composite, and 3.3% for their synthesized material. Although these results are similar to the two sample sets that degraded poorly during composting, their average result of 2.0 ± 1.3% differs by a factor of ~12 when compared to the average degradation of the other samples in this work. This indicates that there may be different rates of degradation for polyether-based polyurethanes when compared to polyester-based ones, and that the bulk properties of the materials may also play a role.

**Figure 2:**
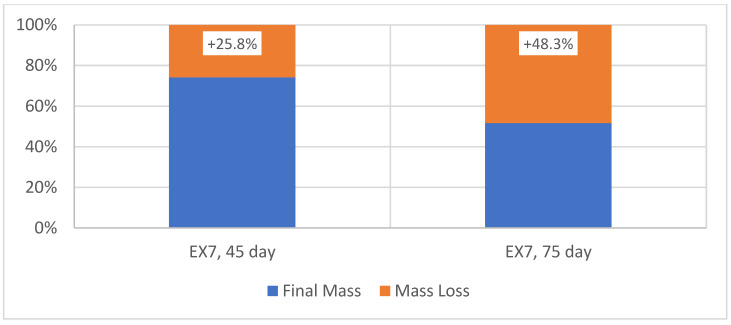
Aggregate sample mass loss from the extended ISO 20200 degradation trial. Samples were handled in accordance with ISO 20200 for 45 and 75 days. The lower, blue portion represents the final mass of the sample as a percentile of mass remaining after the trial. The upper, orange portion and the data callout on the graph represent the percentage mass lost during the trial. Extended trial sample set identifiers can be found in Figure S2 of the Supplementary Materials.

To evaluate the degradation of biobased polyurethane foams over time, an extended trial with endpoints at 45, 75, and 90 days was performed. Of particular interest was the fact that the sample sets of EX7 were prepared from a batch similar to 2 and followed the extended procedure specified in ISO 20200 (Figure 2). The sets of EX7 also contained a probiotic carrier designed to distribute a microbial consortium within the foam bulk and to assist in composting the foam samples; however, the carrier did not contain any bacteria for this specific trial. Although the data were collected for 90 days (Figures S1–S4), excessive fungal growth within and around the sample set led to mass gain, rendering the results at 90 days inconclusive.

Performance-wise, sample **EX7** had similar biodegradability to **3** after a period of 45 days. This indicates that the choice of probiotic carrier provided an additional boost towards polyurethane biodegradation and was in line with the other adduct materials chosen by the polyurethane supplier. With the additional composting time afforded to the 75-day sample of **EX7**, the weight loss increased from 25.8% to 48.3%, resulting in a difference of 22.5% over the additional 30 days. Considering the consistent linear increase in biodegradation rate between the two time periods (R^2^ = 0.9949), there appears to be a constant rate of degradation for this material; however, the inclusion of additional extended trial timepoints would be necessary to determine this outcome.

### 3.2. Test Verification by Thermogravimetric Analysis

To determine whether the results collected during the ISO 20200 trials are valid, there must be a minimum 30% drop in volatile organic solids in the synthetic waste following sample retrieval. This was calculated by performing separate TGA of the synthetic waste samples collected at the end of the trial period and comparing the result against the un-composted initial synthetic waste used as a control. The calculation to determine the decrease in organic volatile solids is as follows, as adapted from the ISO 20200 standard [17].

∆% volatiles = (% control − % sample)/% control × 100%

**Figure 3:**
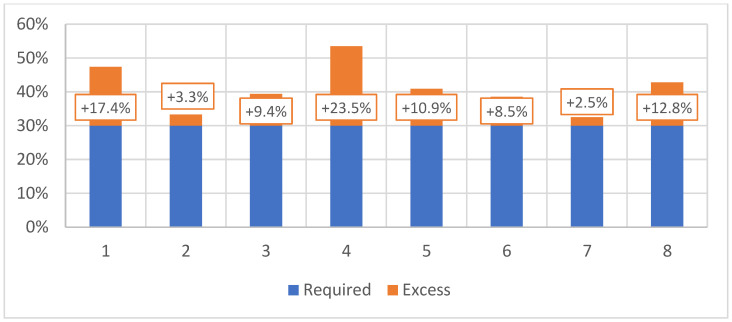
Aggregate decrease in volatile solids content from both ISO 20200 degradation trials. Samples were handled in accordance with ISO 20200 for 45 days. The lower, blue portion represents the required 30% decrease in volatile solids, according to ISO 20200. The upper, orange portion and the data callout on the graph represents the excess percentage volatile organics lost during the trial. Sample set numbers are assigned according to Table 1.

Along with determining whether the material within a composting reactor achieved a sufficient degree of degradation, the data retrieved from the TGA also indicated how active a composting reactor was during the trial. Reasonably, composting reactors that have a larger excess of volatile solid mass loss should also have greater sample degradation if the samples have a similar rate of degradation. In fact, this trend is seen in sample sets **2**–**7**. Alternatively, this indicated that samples **1** and **8** have a slower rate of degradation when compared to the other sets. This also correlates to the visual inspection results of the representative samples in sets **1** and **8**, where there are physical factors that appear to play a role in the degradation rate (Figure 3).

### 3.3. Visual Inspection of Degraded Samples

**Figure 4:**
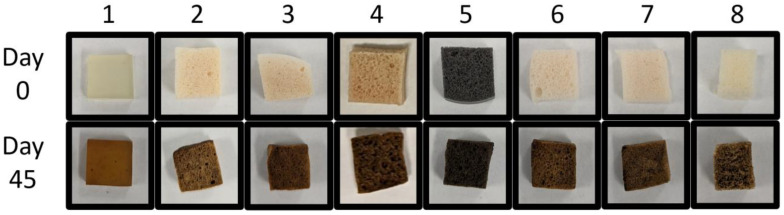
Composite photo image of all representative samples before and after 45 days of simulated composting conditions.

Along with the measured mass loss of each sample, images were also taken to observe representative qualitative changes in the polyurethane materials subjected to degradation. These representative images include photos taken of the sample sets before and after composting, as well as the microscopic images of the surfaces of these materials (Figure 4).

**Figure 5:**
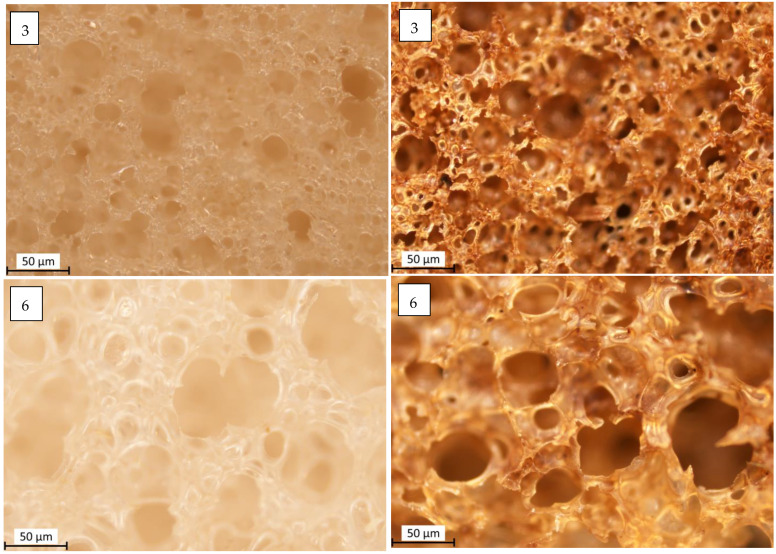
Microscopic image of representative samples of sets **3** (**top**) and **6** (**bottom**), before (**left**) and after (**right**) 45 days of simulated composting conditions. Microscopy was performed at 40× magnification in air.

In general, the two most prominent changes that occurred involved a discoloration from an opaque clear to a patchy brown color and a change from a largely intact cell structure to a fractured one. The change in color appears as the result of staining from the decomposing synthetic waste, which also seems to correlate to the unstained interior of the poorly degraded sample set **8**. As for the change in structure, the surface of the composted samples had more broken cells and jagged edges compared to the un-composted samples, which is an expected result of sample degradation (Figure 5). 

**Figure 6:**
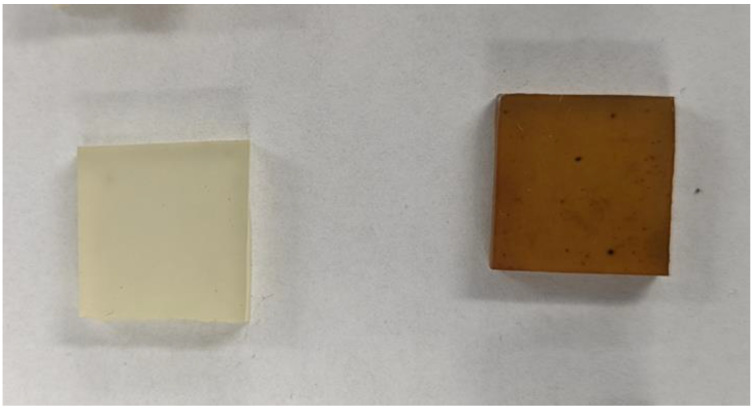
Photo image of a representative of sample set **1** after 45 days of simulated composting conditions. Note that the sample is made of solid and does not have a porous foam structure.

**Figure 7:**
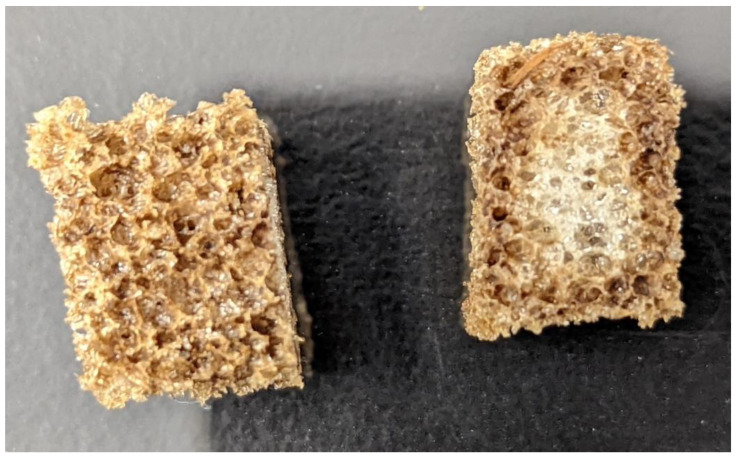
Photo image of a representative of sample set **8** after 45 days of simulated composting conditions. Sample was dissected to show interior of the sample material.

With sample sets **1** and **8** being outliers with low mass loss, additional images were taken of these materials. The observations of these materials suggest that a lack of degradation was caused by poor infiltration of synthetic waste media. For sample **1**, the microscopic images of the commercially obtained biobased thermoplastic polyurethane show a solid material instead of the polyurethane foam structure exhibited by the other sets (Figure 6). With sample **8**, dissecting a representative piece exposes an inner core that retains the original clear/white coloration observed in the sample before degradation (Figure 7). Along with poor infiltration, these images suggest that surface area may play a role in the rate of degradation of biobased polyurethane materials.

### 3.4. Attenuated Total Reflection/Fourier Transform Infrared Spectroscopy (ATR-FTIR)

**Figure 8:**
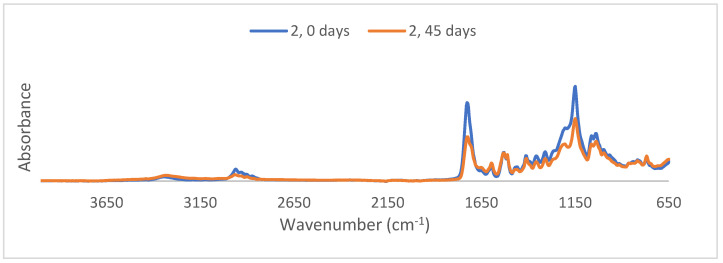
ATR-FTIR of a representative of sample set 2 before and after exposure to simulated composting conditions. Both spectra datasets were linearly scaled for the purpose of comparison, with the peak height at 1524 cm^−1^ as a reference. Note the change in peak height at 1720 and 1150 cm ^− 1^ between the day 0 and day 45 samples, as compared to the intensity of other peaks within the same set.

ATR-FTIR was performed on representatives of each of the sample sets to identify what chemical components were being targeted during degradation. The results from all the samples show that there is a reduction in absorbance at ~1720 cm^−1^ which corresponds to a decrease in the carbonyl C=O stretch, and a reduction at ~1150 cm^−1^ corresponding to a loss of the C-O stretch [21]. There is also an increase in peak intensity at 3300 and 1650 cm^−1^ indicating the appearance of an O-H stretch and a new carbonyl C=O, respectively [19]. Considering that these functional groups appear as part of the polyester linkage within the polyol backbone, the changes in absorbance suggest the degradation of polyester via hydrolysis within the polyurethane (Figure 8). 

Of particular interest is the fact that the ATR-FTIR spectroscopy of sample set 8 demonstrates an uneven transformation of the foam material, where the interior of the sample appears partially preserved compared to the exterior surface. Although the same changes in peak intensity at 3300, 1720, 1650, and 1150 cm^−1^ are observed as for the other polyester-based polyurethane foam samples, the exterior of sample **8** presents the greatest change compared to the spectral analyses of the sample interior and the non-degraded control. (Figure 8) These results correspond to the less-degraded appearance of the interior of sample **8** (Figure 5), suggesting protection via poor infiltration of the degradation media during the ISO20200 trial.

### 3.5. Limitations

Although the ISO 20200 standard focuses on the degradation aspect of the material, this standard is limited in its ability to determine whether the samples have biodegraded into carbon dioxide and nitrates and into other inorganic materials [17]. To determine whether biodegradation has occurred during degradation, ASTM D5338 could be used for this assessment [22]. This standard relies on directly monitoring the release of carbon dioxide from samples within commercially available compost under thermophilic conditions. Although determining the rate of biodegradation of the tested polyurethane materials would be ideal, this standard has the disadvantage of requiring a greater degree of cost and complexity, which make it less ideal for surveying materials with a wide variety of modifications. These costs manifest as specialized equipment for passing moisturized, carbon dioxide-scrubbed air through each of the sample sets, additional controls to determine whether evolved carbon dioxide is coming from the compost or the samples, and carbon dioxide-capturing and monitoring equipment.

Another limitation is that these trials were performed as a survey of a variety of formulations. Although there are trends in mass loss, visual qualities, and ATR-FTIR observations that can be seen between the formulation categories, having single sets of each does impact the statistical significance seen when comparing the individual sets to each other. However, the strength of the data collected appears in the beginning when comparing the results of each of the formulation sets against each other, as well as when taking the average result across the entire set of data. The weakness of the individual statistical power also applies to the results obtained from the TGA analysis, since only a small amount of material can be used to obtain results. To mitigate this, additional care was taken to make sure the compost sets were homogenized before retrieving and running samples for the TGA. Regardless, the purpose of the TGA was to assess whether the composting trials were valid, and an observation of the results obtained mirrors those involving the mass loss of each of the sample sets.

## 4. Conclusions

Considered that most synthetic polymeric materials place a large burden on the environment in terms of petrochemical resource extraction and end-of-use pollution, biobased polyester polyurethanes appear as a path towards a more sustainable alternative. Biobased polyurethanes can be formulated that require fewer petrochemical materials in their production, and the goal of producing a petrochemical-free polymeric material is seen as viably achievable in the near term. For this study, biobased polyester polyurethanes consisting of a poly(1,3-propylene-succinate)-co-methylenediphenyl diisocyanate backbone were subjected to ISO 20200, which evaluated degradation under composting conditions, followed by ATR-FTIR analysis of the degraded foams. Compared to the results obtained by Kupka et al. [19], the biobased polyester-backboned polyurethanes also appear to have an average mass loss of 24.3 ± 4.0% when subjected to composting conditions for 45 days, which suggests that these materials are less likely to persist in the environment when compared to other types of commodity plastics. [23] Additionally, the disappearance of FTIR peaks at 3300, 1720, 1650, and 1150 cm^−1^ in degraded foams suggests enzymatic degradation of key backbone polymeric linkages. [8,9] Ideally, society will benefit from the development and use of bio-sustainable polymeric materials, and additional testing and evaluation of these materials will contribute to that goal.

## Figures and Tables

**Table 1:** List of samples for ISO 20200 degradation trials. Sample 1 is a commercial polyether control.

#	Polyol	Bio-Surfactant	Lignin	Charcoal	Bio-Isocyanate	Glycerol Crosslinker	Evoco Probiotics
**1**	Polyether	CONTROL SET					
**2**	Polyester	Tributyl citrate	No	No	No	Normal	1%
**3**	Polyester	Triethyl citrate	No	No	No	Normal	1%
**4**	Polyester	Tributyl citrate	Yes	No	No	Normal	1%
**5**	Polyester	Tributyl citrate	Yes	Yes	No	Normal	1%
**6**	Polyester	Tributyl citrate	No	No	30%	Normal	1%
**7**	Polyester	Tributyl citrate	No	No	30%	Normal	1%
**8**	Polyester	Tributyl citrate	No	No	No	2×	Yes

## Data Availability

The original contributions presented in the study are included in the article/Supplementary Material, further inquiries can be directed to the corresponding author.

## Supplementary Materials

The following supporting information can be downloaded at: https://www.mdpi.com/article/10.3390/polym16142071/s1, Figure S1: Tabulated degradation data for ISO 20200 degradation trial.; Figure S2: List of extended ISO 20200 degradation trial samples (EX); Figure S3: Tabulated degradation data for extended ISO 20200 degradation trial samples (EX;. Figure S4: Graph representing data from Figure S3; Figure S5: Composite image of extended ISO 20200 degradation trial samples (EX); Figure S6: Raw FTIR data for EX1 at 0 days and 45 days; Figure S7: Raw FTIR data for EX2 at 0 days and 45 days; Figure S8: Raw FTIR data for EX3 at 0 days (top) and 45 days; Figure S9: Raw FTIR data for EX4 at 0 days (top) and 45 days; Figure S10: Raw FTIR data for EX5 at 0 days and 45 days; Figure S11: Raw FTIR data for EX6 at 0 days (top) and 45 days; Figure S12: Raw FTIR data for EX7 at 0 days (top) and 45 days.
