# Supplementary material for: Degradation of Bioderived Polyurethane Composites by Spectroscopy in ISO20200 Composting Conditions
Source: Polymers (Basel). 2024 Jul 20;16(14):2071. doi: 10.3390/polym16142071 (PMC11280820; doi:10.3390/polym16142071)

## Supplemental information for

# Degradation of Bioderived Polyurethane Polyester Composites by Spectroscopy in ISO20200 Composting Conditions.

Alexander Caschera[1,2], Tristan Calayan[1], Nicola Piccolo[1], Adel Kakroodi[2], Jason James Robinson[2] and Guerino Sacripante[1*]

[1] *Department of Chemistry & Biology, Toronto Metropolitan University, 350 Victoria Street, Toronto ON M5B 2K3 Canada*

[2] *Evoco Ltd, 661 University Ave., Toronto, ON M5G 1L7*

**Figure S1** – Tabulated degradation data for ISO 20200 degradation trial. .......... 2
**Figure S2** – List of extended ISO 20200 degradation trial samples (EX). Note that each set of samples were kept in the same composting reactor. Data for 90 day degradation is considered to be inaccurate due to mold growth on samples leading to artificial mass gain. .......... 2
**Figure S3** – Tabulated degradation data for extended ISO 20200 degradation trial samples (EX). Note that each set of samples were kept in the same composting reactor. Data for 90 day degradation is considered to be inaccurate due to mold growth on samples leading to artificial mass gain. .......... 2
**Figure S4** – Graph representing data from Figure S3. .......... 3
**Figure S5** – Composite image of extended ISO 20200 degradation trial samples (EX). .......... 4
**Figure S6** – Raw FTIR data for EX1 at 0 days (top) and 45 days (bottom) .......... 5
**Figure S7** – *Raw FTIR data for EX2 at 0 days (top) and 45 days (bottom)* .......... 6
**Figure S8** – *Raw FTIR data for EX3 at 0 days (top) and 45 days (bottom)* .......... 7
**Figure S9** – *Raw FTIR data for EX4 at 0 days (top) and 45 days (bottom)* .......... 8
**Figure S10** – *Raw FTIR data for EX5 at 0 days (top) and 45 days (bottom)* .......... 9
**Figure S11** – *Raw FTIR data for EX6 at 0 days (top) and 45 days (bottom)* .......... 10
**Figure S12** – *Raw FTIR data for EX7 at 0 days (top) and 45 days (bottom)* .......... 11

**Figure S1** – Tabulated degradation data for ISO 20200 degradation trial.

| # | Compost period | Initial Mass (g) | Final Mass (g) | Mass Loss (g) | Mass Loss (%) |
|---|---|---|---|---|---|
| ***1*** | 45 days | 10.591 | 10.346 | 0.245 | 2.3 |
| ***2*** | 45 days | 10.129 | 8.122 | 2.007 | 19.8 |
| ***3*** | 45 days | 10.237 | 7.591 | 2.646 | 25.8 |
| ***4*** | 45 days | 10.037 | 6.939 | 3.098 | 30.9 |
| ***5*** | 45 days | 10.232 | 7.477 | 2.755 | 26.9 |
| ***6*** | 45 days | 10.270 | 7.745 | 2.525 | 24.6 |
| ***7*** | 45 days | 10.341 | 7.831 | 2.510 | 24.3 |
| ***8*** | 45 days | 10.135 | 9.932 | 0.203 | 2.0 |

**Figure S2** – List of extended ISO 20200 degradation trial samples (EX). Note that each set of samples were kept in the same composting reactor. Data for 90 day degradation is considered to be inaccurate due to mold growth on samples leading to artificial mass gain.

| # | Polyol | Surfactant | Lignin adduct | Charcoal adduct | Bio-based isocyanate | Glycerol Crosslinker | Evoco Probiotics |
|---|---|---|---|---|---|---|---|
| ***EX1*** | Polyester | Tributyl citrate | No | No | No | Normal | No |
| ***EX2*** | Polyester (GMR-38 polyol) | Tributyl citrate | No | No | No | Normal | No |
| ***EX3*** | Polyester (GMR-45 polyol) | Tributyl citrate | No | No | No | Normal | No |
| ***EX4*** | Polyester | Tributyl citrate | No | No | No | Normal | **0.5%** |
| ***EX5*** | Polyester | Tributyl citrate | No | No | No | Normal | **1%** |
| ***EX6*** | Polyester | **Tween** | No | No | No | Normal | No |
| ***EX7*** | Polyester | Tributyl citrate | No | No | No | Normal | **5% (carrier only)** |

**Figure S3** – Tabulated degradation data for extended ISO 20200 degradation trial samples (EX). Note that each set of samples were kept in the same composting reactor. Data for 90 day degradation is considered to be inaccurate due to mold growth on samples leading to artificial mass gain.

| # | Compost period | Initial Mass (g) | Final Mass (g) | Mass Loss (g) | Mass Loss (%) |
|---|---|---|---|---|---|
| ***EX1*** | 45 days | 5.11 | 4.00 | 1.11 | 21.7 |
| | 75 days | 5.34 | 4.33 | 1.01 | 18.9 |
| | 90 days | 5.40 | 3.84 | 1.56 | 28.9 |
| ***EX2*** | 45 days | 5.44 | 4.00 | 1.44 | 26.5 |

| | | | | | |
|---|---|---|---|---|---|
| | 75 days | 5.25 | 3.16 | 2.09 | 39.8 |
| | 90 days | 5.34 | 3.52 | 1.82 | 34.1 |
| ***EX3*** | 45 days | 5.32 | 4.11 | 1.21 | 22.7 |
| | 75 days | 5.34 | 4.01 | 1.33 | 24.9 |
| | 90 days | 5.28 | 3.64 | 1.64 | 31.1 |
| ***EX4*** | 45 days | 5.31 | 3.89 | 1.42 | 26.7 |
| | 75 days | 5.20 | 3.44 | 1.76 | 33.8 |
| | 90 days | 5.28 | 3.12 | 2.16 | 40.9 |
| ***EX5*** | 45 days | 5.14 | 3.84 | 1.30 | 25.3 |
| | 75 days | 5.26 | 4.13 | 1.13 | 21.5 |
| | 90 days | 5.27 | 3.54 | 1.73 | 32.8 |
| ***EX6*** | 45 days | 5.30 | 3.98 | 1.32 | 24.9 |
| | 75 days | 5.44 | 3.30 | 2.14 | 39.3 |
| | 90 days | 5.31 | 3.55 | 1.76 | 33.1 |
| ***EX7*** | 45 days | 5.24 | 3.89 | 1.35 | 25.8 |
| | 75 days | 5.28 | 2.73 | 2.55 | 48.3 |
| | 90 days | 5.37 | 2.98 | 2.39 | 44.5 |

**Figure S4** – Graph representing data from Figure S3.

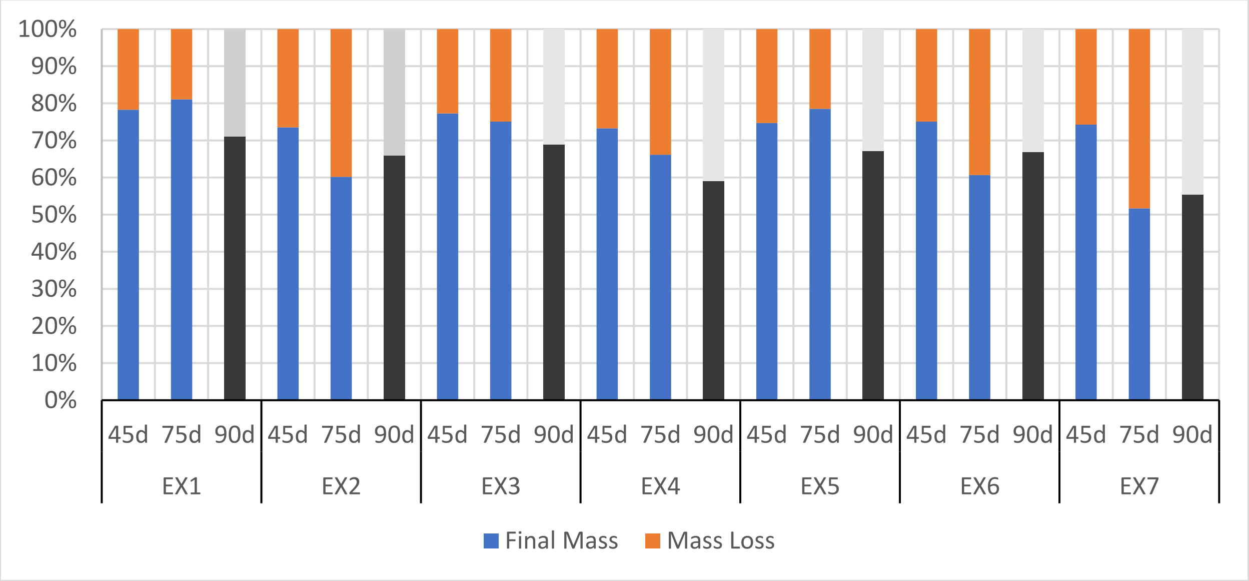

**Figure S5** – Composite image of extended ISO 20200 degradation trial samples (EX).

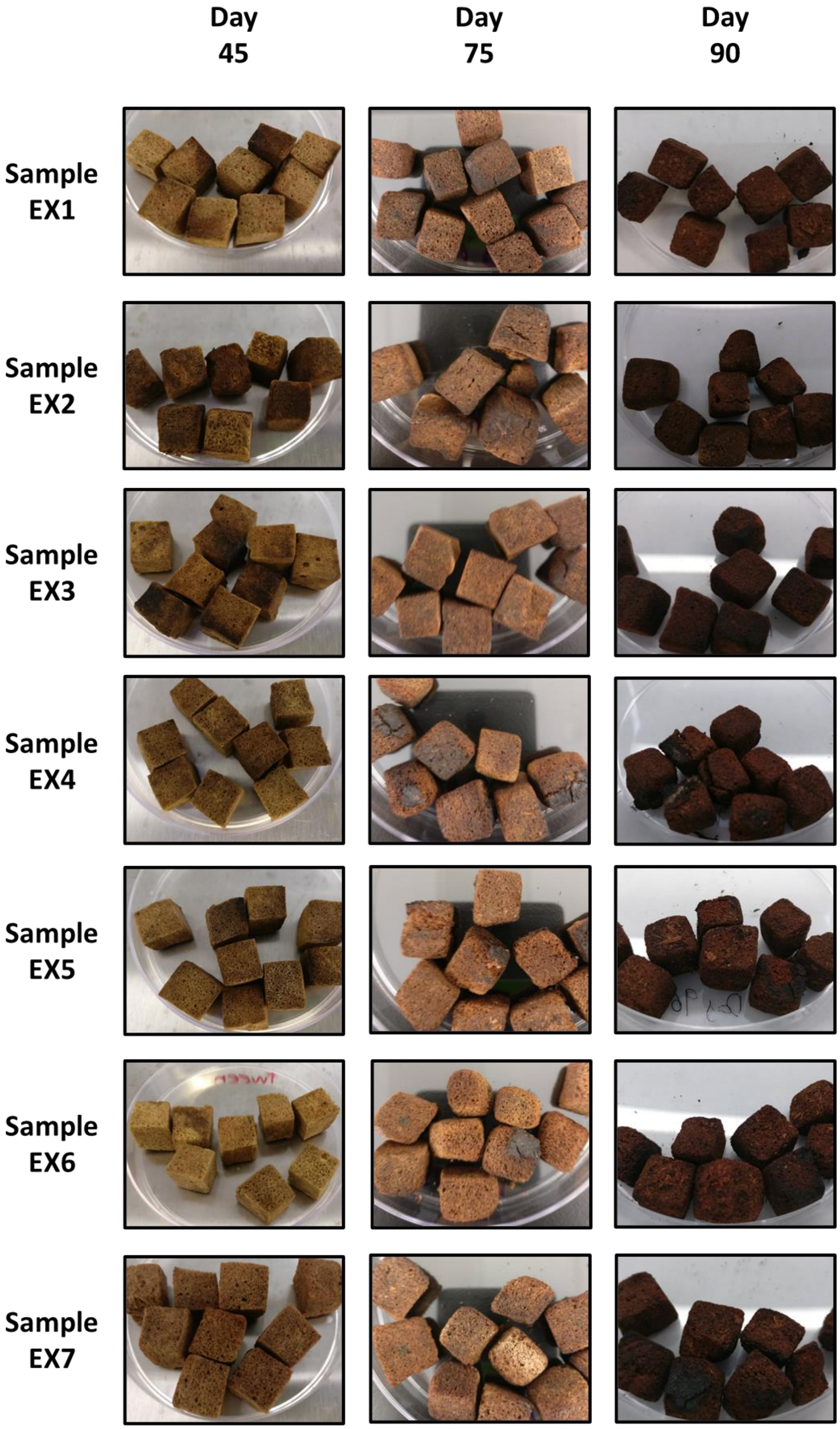

**Figure S6** – Raw FTIR data for EX1 at 0 days (top) and 45 days (bottom)

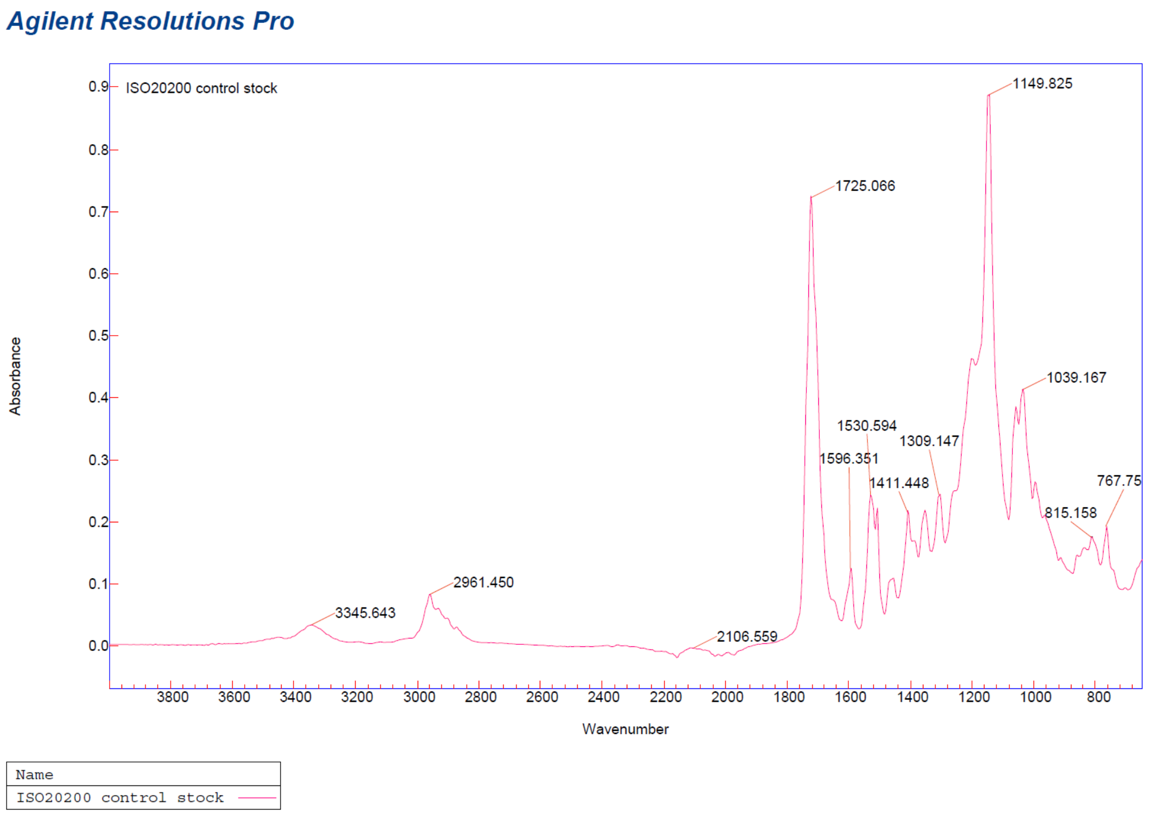


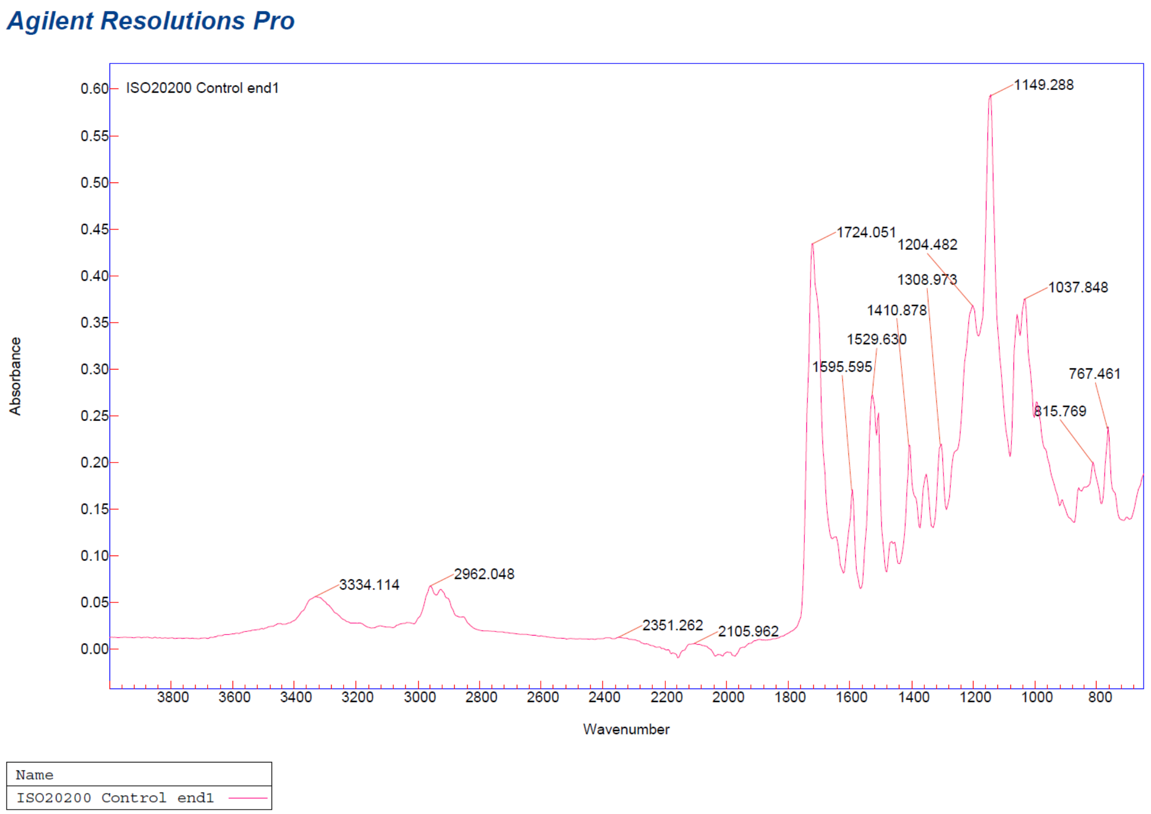

**Figure S7** – *Raw FTIR data for EX2 at 0 days (top) and 45 days (bottom)*

## Agilent Resolutions Pro

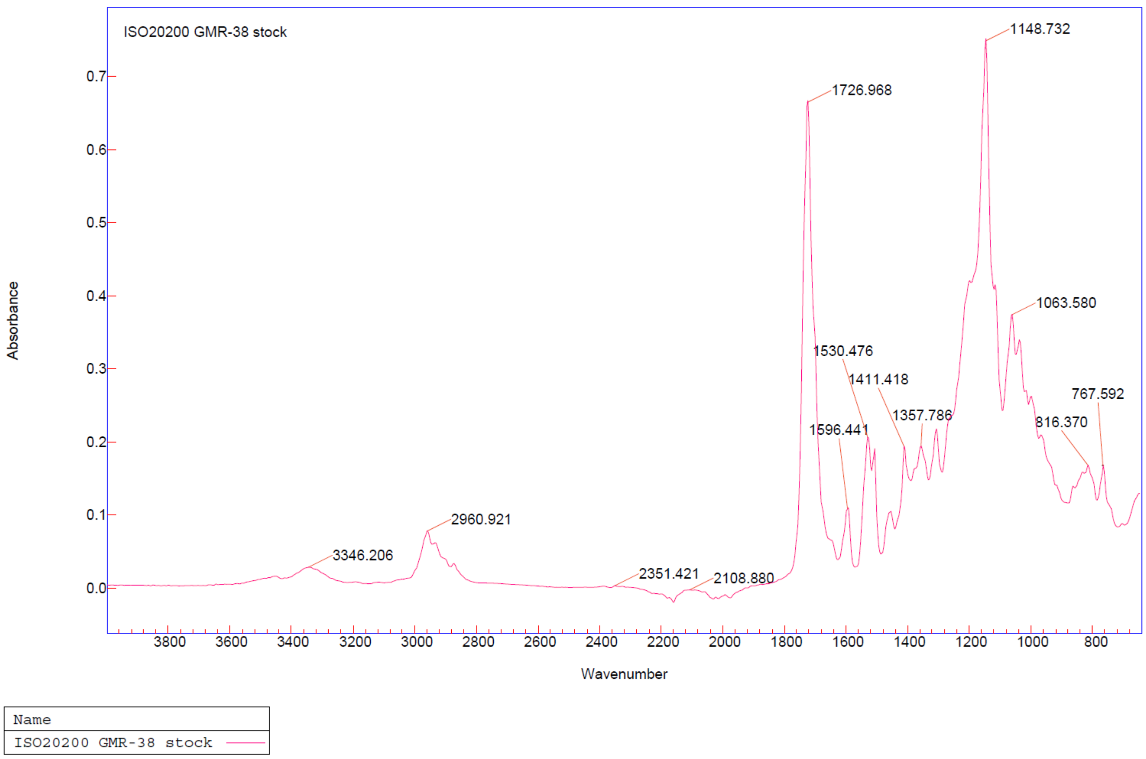


## Agilent Resolutions Pro

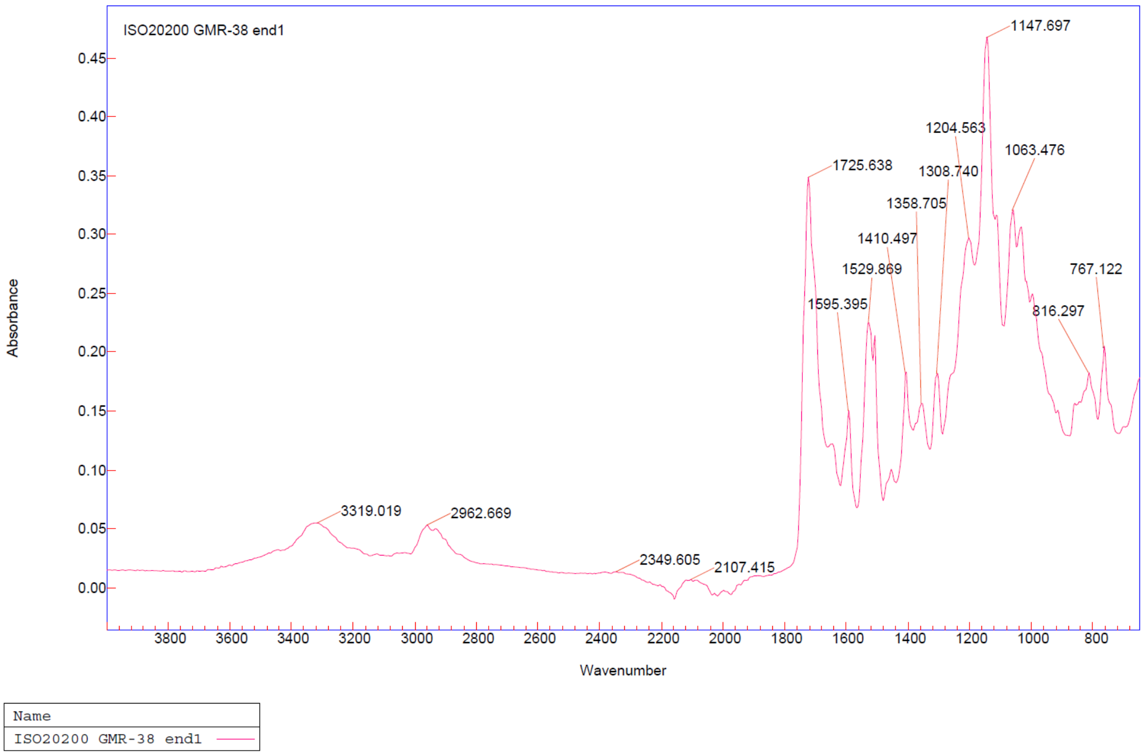

**Figure S8** – *Raw FTIR data for EX3 at 0 days (top) and 45 days (bottom)*

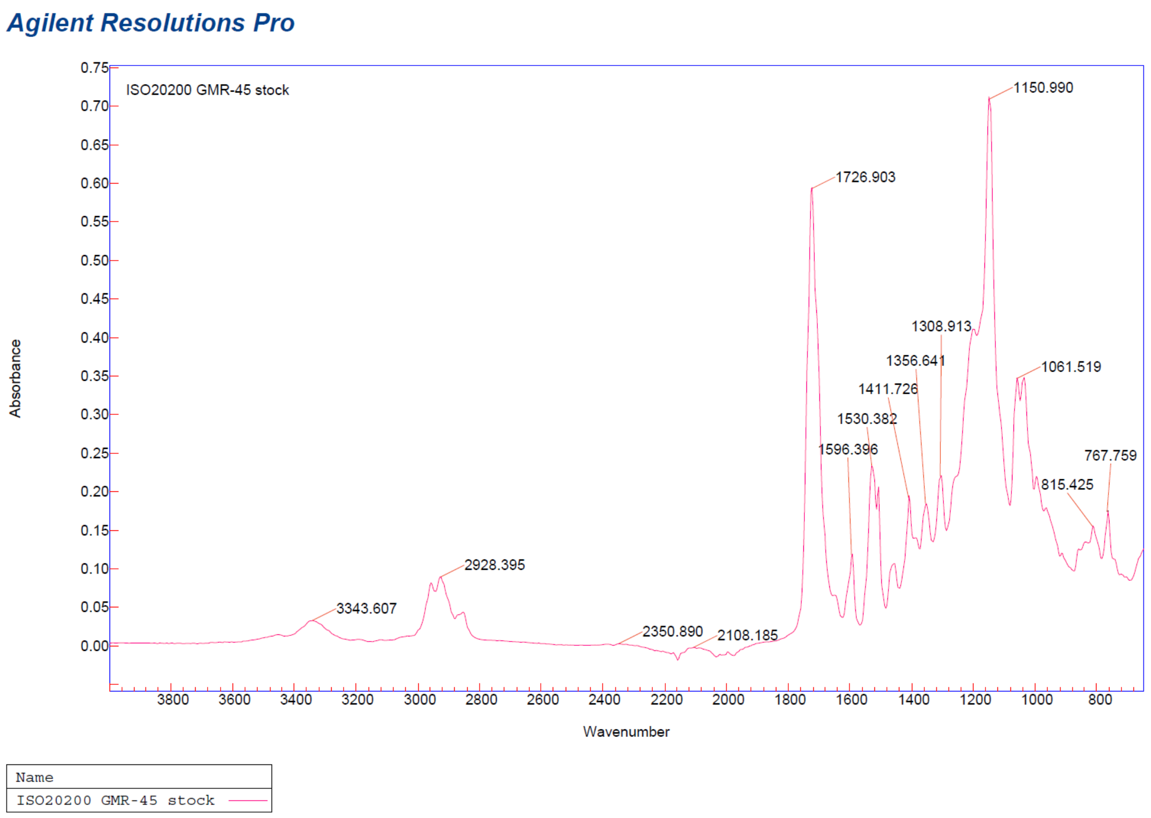


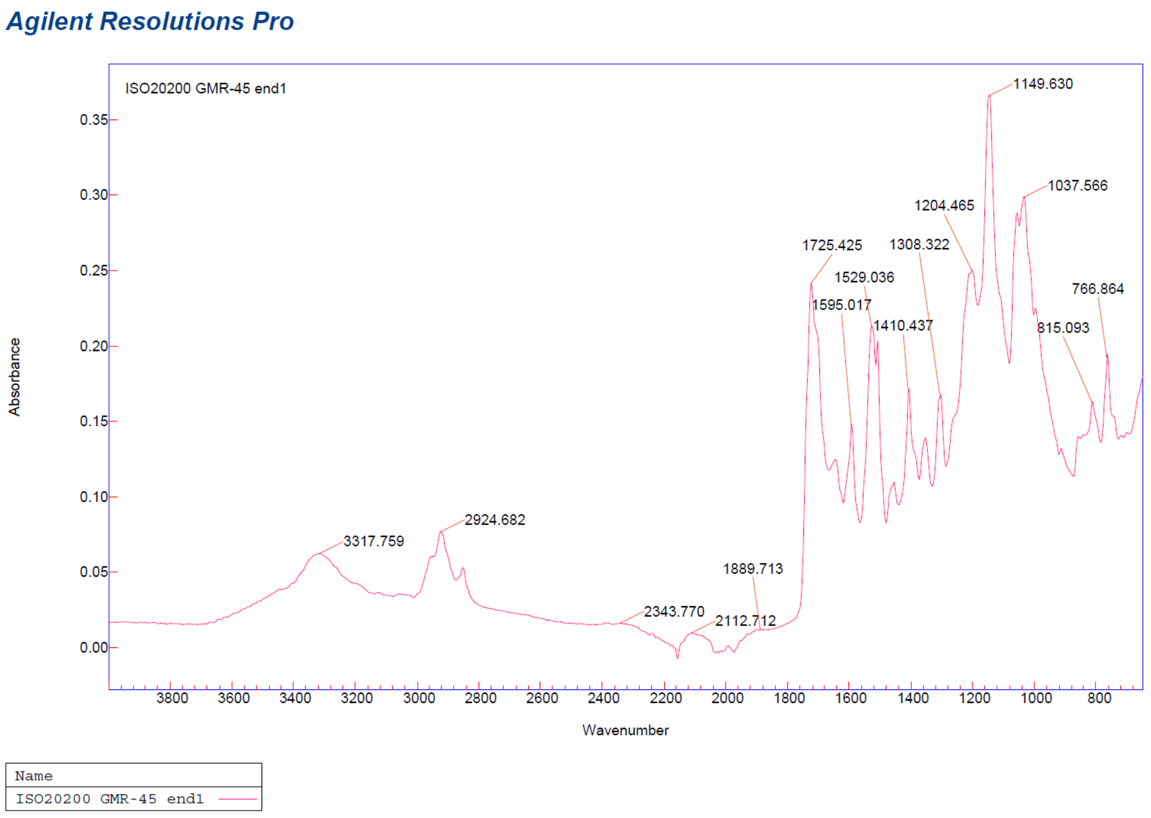

**Figure S9** – *Raw FTIR data for EX4 at 0 days (top) and 45 days (bottom)*

## Agilent Resolutions Pro

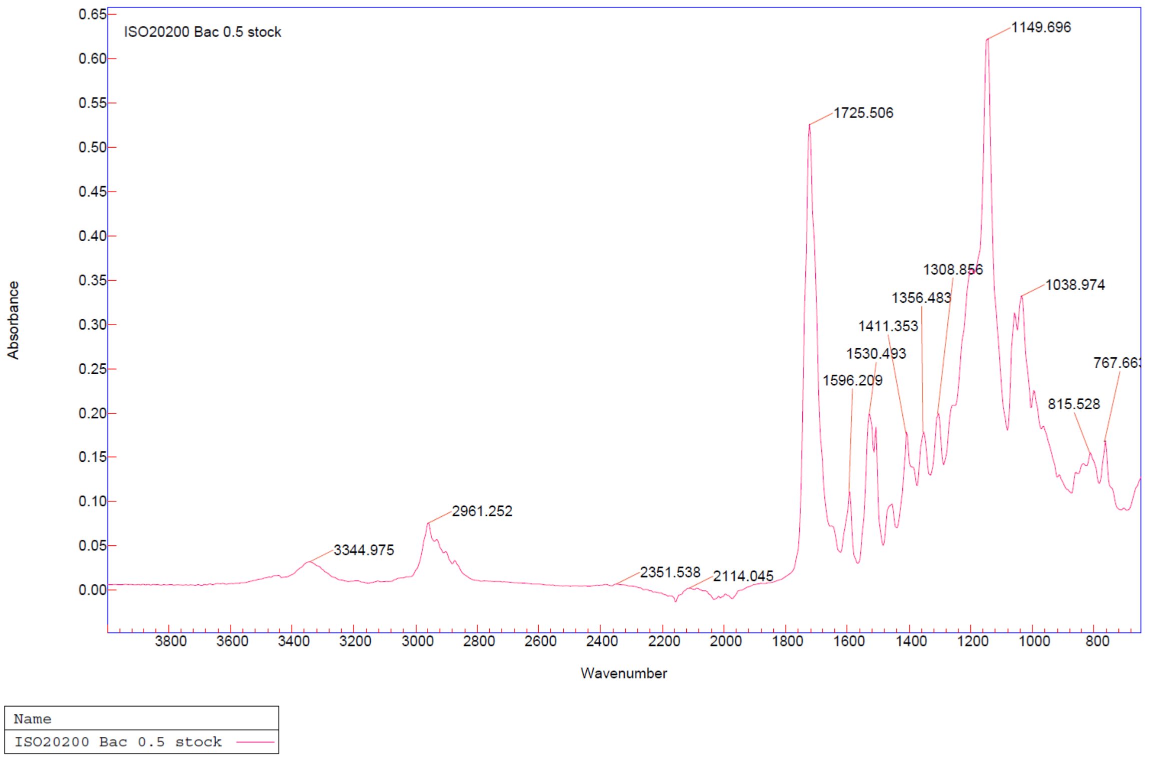


## Agilent Resolutions Pro

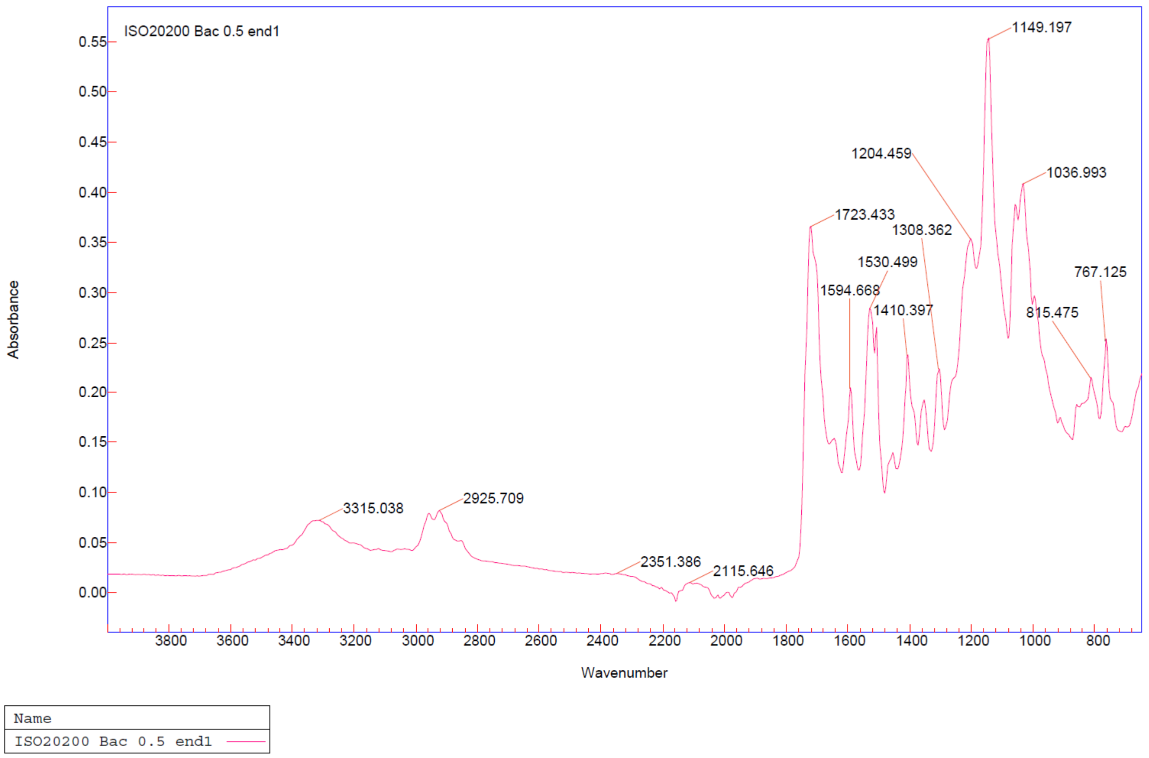

**Figure S10** – *Raw FTIR data for EX5 at 0 days (top) and 45 days (bottom)*

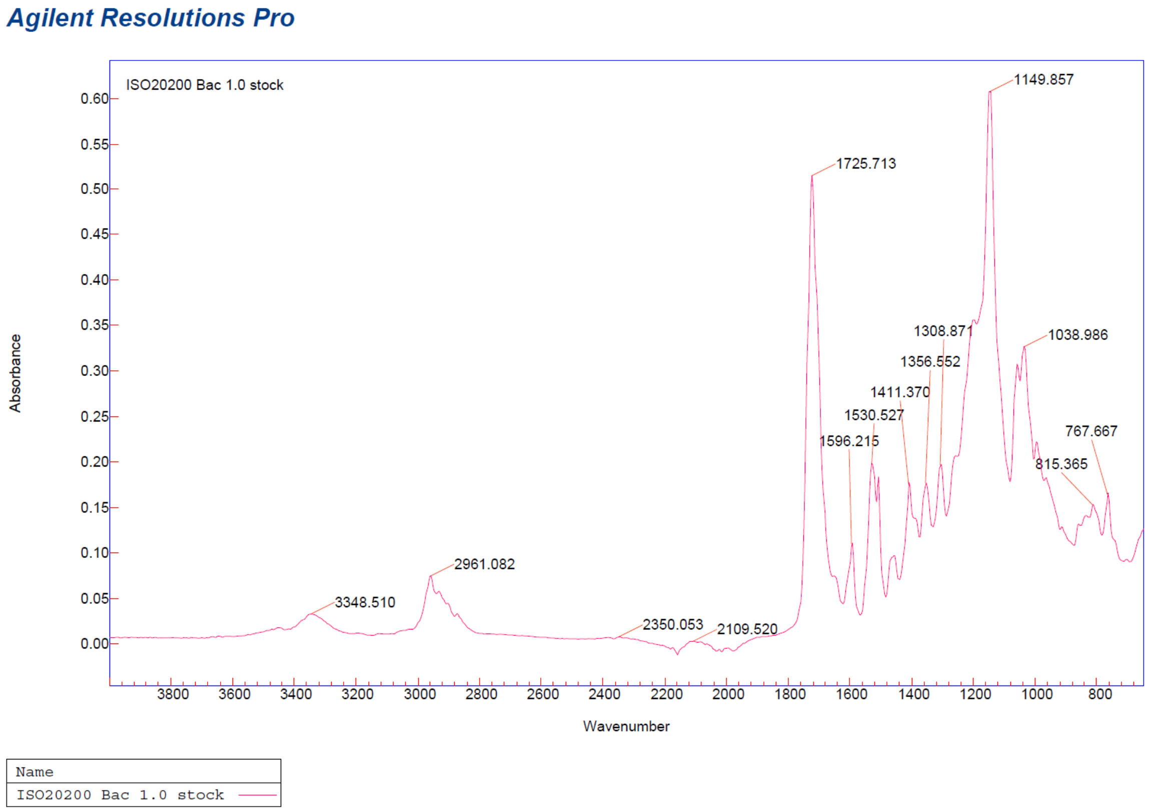


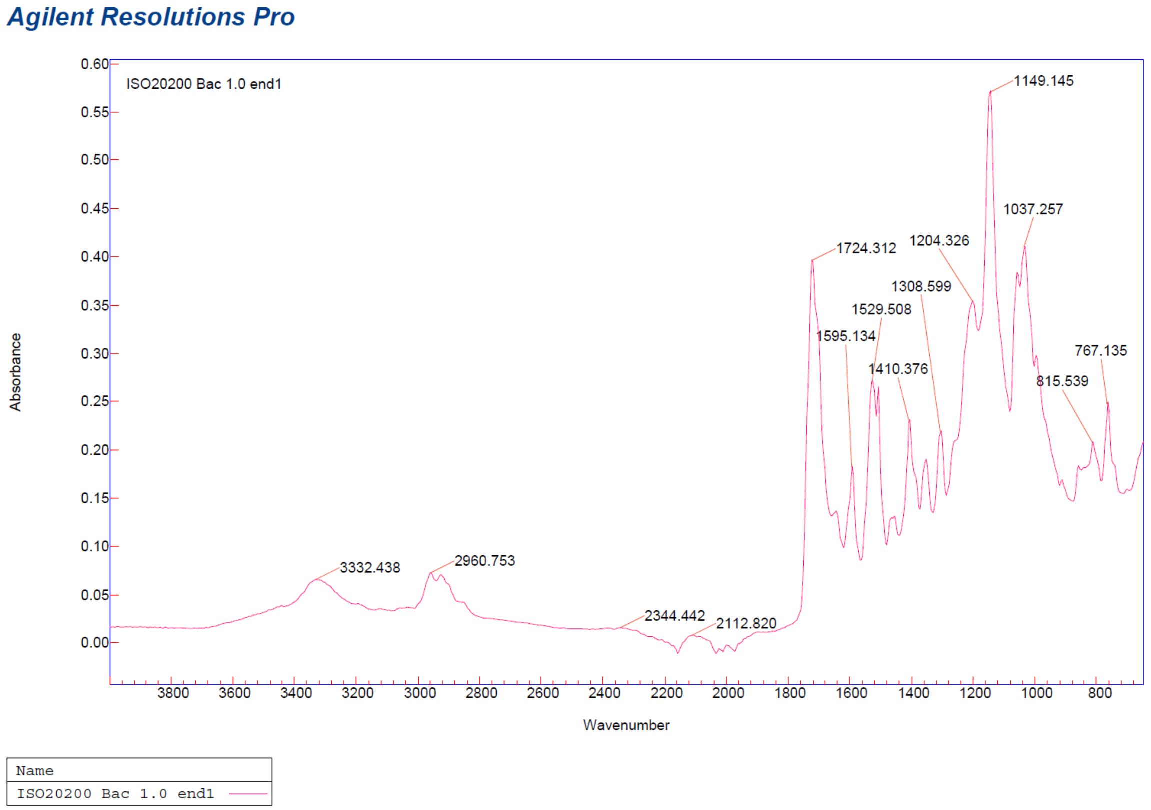

**Figure S11** – *Raw FTIR data for EX6 at 0 days (top) and 45 days (bottom)*

## Agilent Resolutions Pro

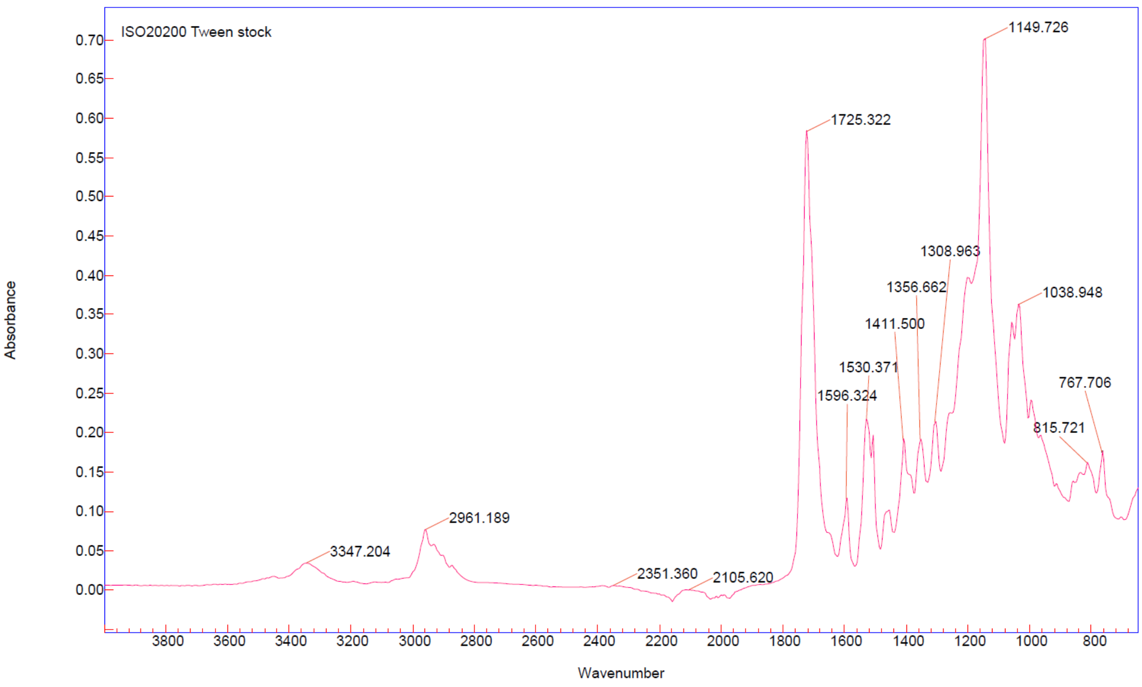


| Name |
|---|
| ISO20200 Tween stock |

## Agilent Resolutions Pro

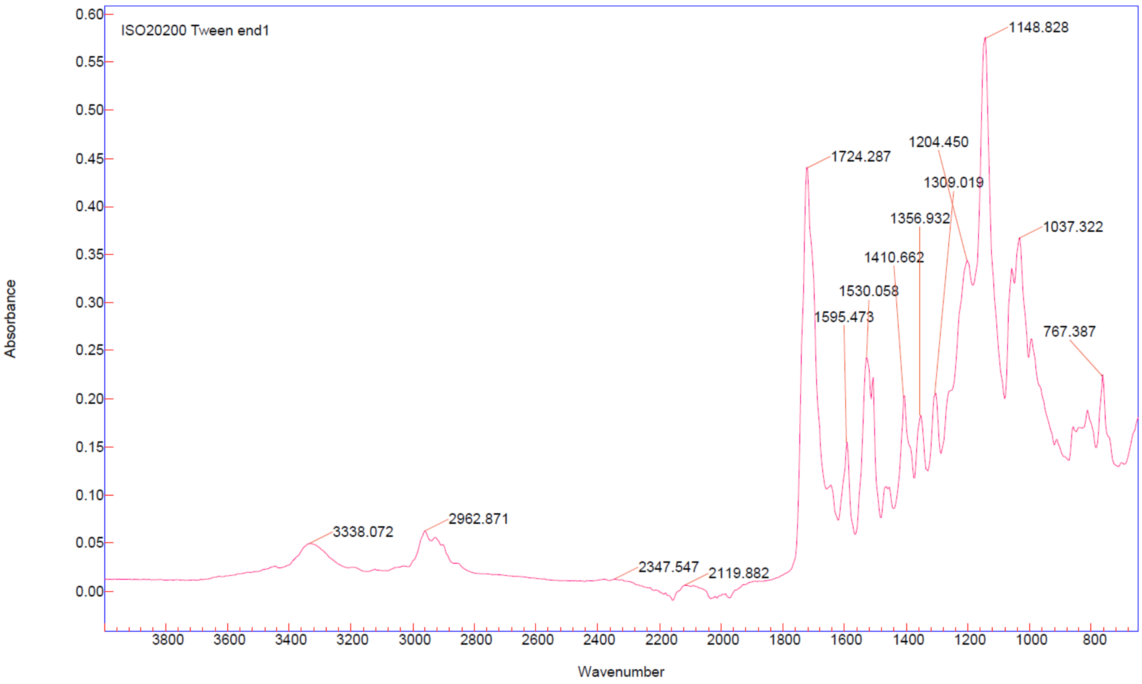


| Name |
|---|
| ISO20200 Tween end1 |

**Figure S12** – *Raw FTIR data for EX7 at 0 days (top) and 45 days (bottom)*

## Agilent Resolutions Pro

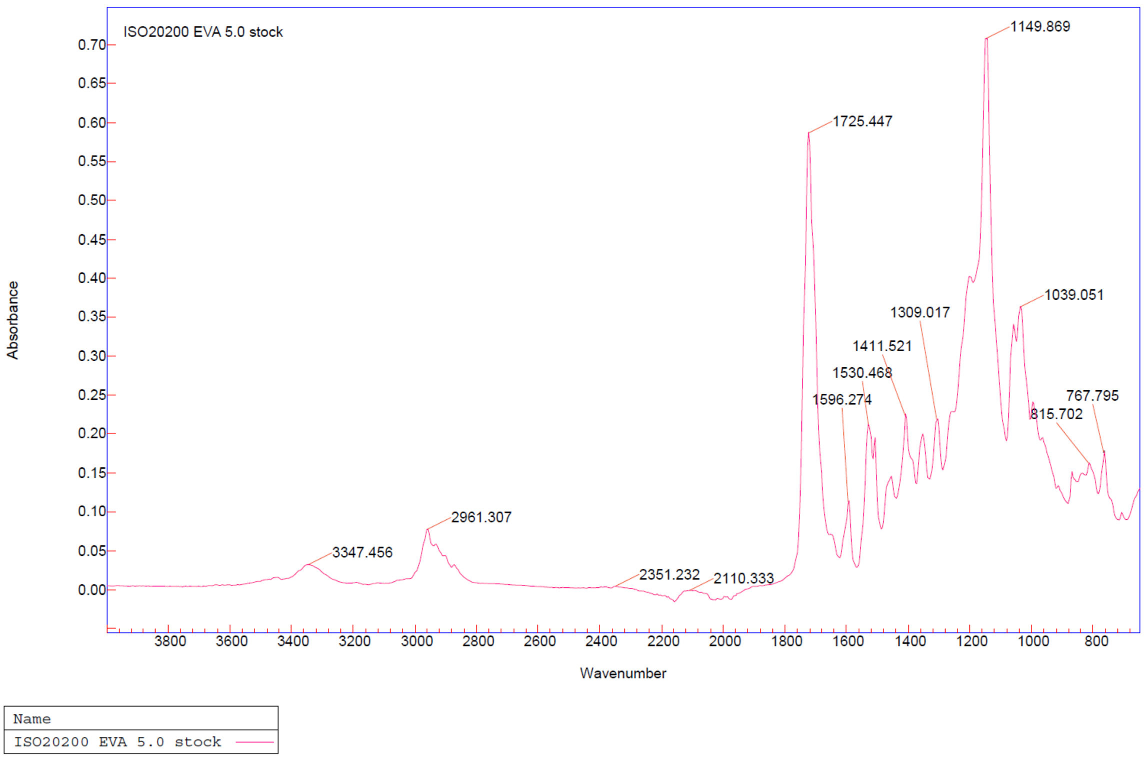


## Agilent Resolutions Pro

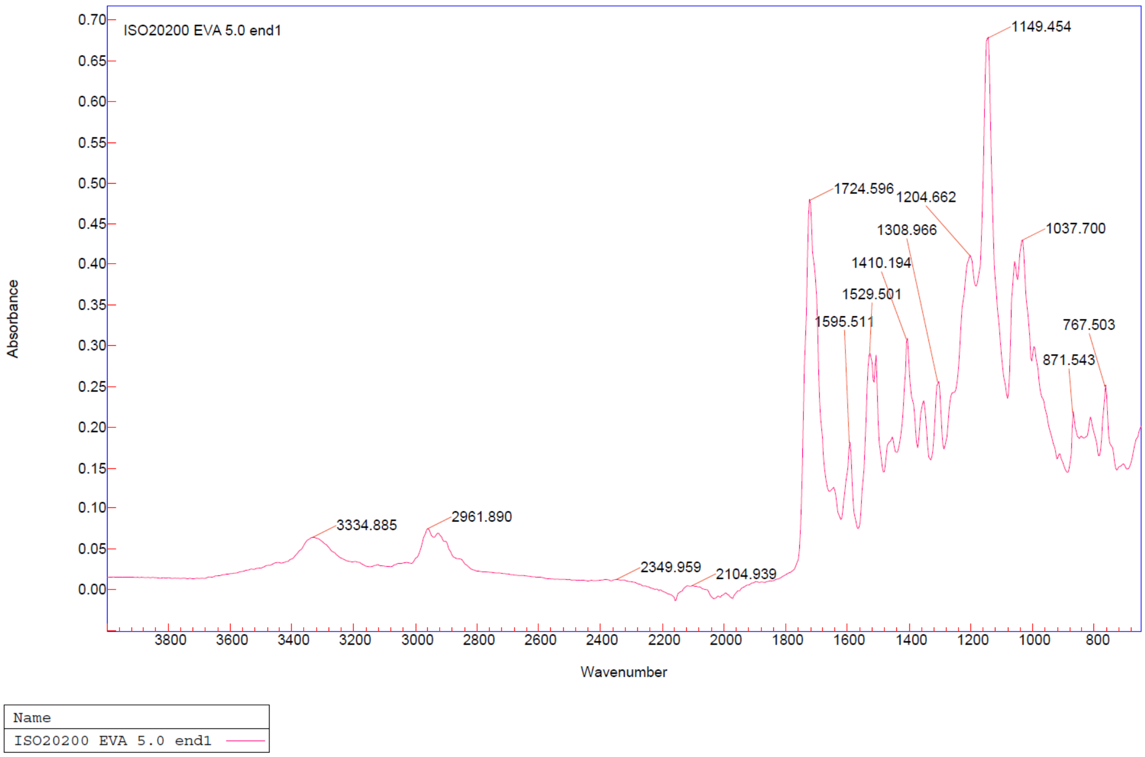

Supplement: Supplementary file 1 [file polymers-16-02071-s001.zip › polymers-2961514-supplementary.pdf]
